# Specific subpopulations of hypothalamic leptin receptor-expressing neurons mediate the effects of early developmental leptin receptor deletion on energy balance

**DOI:** 10.1016/j.molmet.2018.06.001

**Published:** 2018-06-06

**Authors:** Alan C. Rupp, Margaret B. Allison, Justin C. Jones, Christa M. Patterson, Chelsea L. Faber, Nadejda Bozadjieva, Lora K. Heisler, Randy J. Seeley, David P. Olson, Martin G. Myers

**Affiliations:** 1 Department of Internal Medicine, University of Michigan Medical School, Ann Arbor, MI, USA; 2 Department of Surgery, University of Michigan Medical School, Ann Arbor, MI, USA; 3 Rowett Institute, University of Aberdeen, Aberdeen, UK; 4 Department of Pediatrics and Communicable Diseases, University of Michigan Medical School, Ann Arbor, MI, USA

**Keywords:** leptin receptor, arcuate nucleus, DMH, obesity, cre recombinase, ghrh, htr2c

## Abstract

**Objective:**

To date, early developmental ablation of leptin receptor (LepRb) expression from circumscribed populations of hypothalamic neurons (e.g., arcuate nucleus (ARC) *Pomc*- or *Agrp*-expressing cells) has only minimally affected energy balance. In contrast, removal of LepRb from at least two large populations (expressing *vGat* or *Nos1*) spanning multiple hypothalamic regions produced profound obesity and metabolic dysfunction. Thus, we tested the notion that the total number of leptin-responsive hypothalamic neurons (rather than specific subsets of cells with a particular molecular or anatomical signature) subjected to early LepRb deletion might determine energy balance.

**Methods:**

We generated new mouse lines deleted for LepRb in ARC *Ghrh*^*Cre*^ neurons or in *Htr2c*^*Cre*^ neurons (representing roughly half of all hypothalamic LepRb neurons, distributed across many nuclei). We compared the phenotypes of these mice to previously-reported models lacking LepRb in *Pomc*, *Agrp*, *vGat* or *Nos1* cells.

**Results:**

The early developmental deletion of LepRb from *vGat* or *Nos1* neurons produced dramatic obesity, but deletion of LepRb from *Pomc*, *Agrp*, *Ghrh*, or *Htr2c* neurons minimally altered energy balance.

**Conclusions:**

Although early developmental deletion of LepRb from known populations of ARC neurons fails to substantially alter body weight, the minimal phenotype of mice lacking LepRb in *Htr2c* cells suggests that the phenotype that results from early developmental LepRb deficiency depends not simply upon the total number of leptin-responsive hypothalamic LepRb cells. Rather, specific populations of LepRb neurons must play particularly important roles in body energy homeostasis; these as yet unidentified LepRb cells likely reside in the DMH.

## Highlights

• Mice lacking LepRb in Ghrh neurons exhibit no alterations in energy balance or growth.
• Mice lacking LepRb in Htr2c neurons (∼50% of hypothalamic LepRb neurons) are not obese.
• Specific subsets of LepRb neurons play particularly important roles in body energy homeostasis.
• LepRb cells crucial for the control of energy balance likely lie in the DMH.

## 1 Introduction

Obesity, which affects more than 1/3 of people in developed countries, predisposes to diabetes, cardiovascular disease, and other serious comorbidities [1]. To design effective treatments for obesity, we must first understand the systems that control energy balance and which represent potential therapeutic targets. The hormone leptin, which is produced by adipose tissue to signal the repletion of fat stores, acts via its receptor (LepRb) on hypothalamic neurons to suppress food intake and permit energy expenditure [2]. Leptin- or LepRb-deficient humans and rodent models display dramatic hyperphagia and reduced energy expenditure, leading to severe obesity [3], [4], [5]. Thus, the hypothalamic neurons by which leptin mediates the control of energy balance represent important controllers of energy balance.

### 1.1 Hypothalamic LepRb neurons

Within the hypothalamus, the arcuate nucleus (ARC), ventromedial hypothalamic nucleus (VMN), dorsomedial hypothalamus (DMH), lateral hypothalamic area (LHA), and ventral premammillary nucleus (PMv) contain substantial numbers of LepRb neurons [6]. While roles for many molecularly-defined and anatomically-circumscribed populations of LepRb have been examined, the early developmental deletion of LepRb from these previously-studies populations has not resulted in obesity similar to the severe obesity observed in entirely LepRb-deficient *db/db* mice [7], [8], [9], [10], [11]. Indeed, while orexigenic ARC neurons that contain agouti-related peptide (AgRP), neuropeptide Y (NPY) and gamma amino butyric acid (GABA) (AgRP neurons) and anorexigenic ARC proopiomelanocortin (POMC)-containing neurons each express LepRb and play crucial roles in energy balance, early developmental ablation of LepRb from AgRP and/or POMC neurons minimally alters energy balance [7], [8].

Similarly, manipulation of LepRb expression in other circumscribed sets of LepRb neurons examined to date (e.g., neurotensin (*Nts*) neurons of the LHA, steroidogenic factor-1 (*Sf1*) neurons in the VMN) minimally impacts energy balance [10], [11]. In contrast, LepRb deletion from some large, widely-distributed hypothalamic populations results in profound obesity and hyperphagia. Indeed, deletion of LepRb in *vGat* (*Slc32a1*)-expressing GABAergic neurons (representing ∼50% of hypothalamic LepRb neurons, including the majority of LepRb neurons in the DMH and LHA, along with AgRP cells and other ARC neurons) or *Nos1* neurons (representing ∼25% of hypothalamic LepRb neurons, including the majority of PMv neurons plus smaller numbers of LepRb cells in the ARC, DMH, VMN and LHA) promotes dramatic obesity [12], [13].

### 1.2 Populations of LepRb neurons that are or are not subject to compensation following early developmental deletion

Early alterations in AgRP neurons (e.g., neuron ablation or LepRb deletion) are compensated during postnatal development, while alterations in mature AgRP neurons in adults have profound effects on leptin action and energy balance [7], [14], [15], [16]. Indeed, while the deletion of LepRb neurons from adult AgRP neurons provokes dramatic obesity, the early developmental deletion of LepRb from AgRP neurons produces little metabolic derangement. Thus, while leptin action on AgRP neurons in adults plays important roles in energy balance, the lack of direct leptin action on AgRP neurons is unlikely to contribute substantially to the phenotype of entirely LepRb-null *db/db* mice. Hence, additional (non-AgRP) LepRb neurons that are not subject to developmental compensation must underlie the majority of the *db/db* phenotype and play important roles in leptin action. In this manuscript, we employ multiple models that mediate the early developmental deletion of LepRb in subpopulations of hypothalamic neurons to define sets of LepRb neurons that mediate leptin action and in which the loss of LepRb is not developmentally compensated (unlike AgRP neurons).

### 1.3 Potential types of LepRb neurons that control energy balance

This non-developmentally compensated control of energy balance might be distributed across multiple hypothalamic nuclei and cell types, with many types of LepRb neurons contributing similarly to the control of energy balance, such that the ablation of leptin action in a threshold number of LepRb neurons (without regard to their location or identity) disrupts the control of energy balance. It is also possible that small, but currently unidentified, populations of LepRb neurons mediate the main effect of leptin/LepRb on energy balance that is not subject to developmental compensation.

Here, we test these possibilities by studying mice deleted for LepRb in previously unexamined sets of growth hormone-releasing hormone-Cre (*Ghrh*^*Cre*^) LepRb neurons of the ARC (LepRb^Ghrh^ cells) as well as serotonin receptor 2c (*Htr2c*)-expressing LepRb (LepRb^Htr2c^) neurons that lie mainly in the PMv, VMH, and LHA (a few also lie in the ARC and DMH). We examined these mouse strains together with mice subjected to early developmental deletion of LepRb in *Agrp*, *Pomc*, *vGat*, and *Nos1* neurons. Our results and analysis suggest that small (and as yet unidentified) populations of DMH LepRb neurons that are not subject to developmental compensation likely play crucial roles in the control of energy balance by leptin.

## 2 Results

### 2.1 The role for *Ghrh*^*Cre*^ neurons in leptin action

To identify novel subpopulations of hypothalamic LepRb neurons, we previously performed translational profiling of their transcriptome using translating ribosome affinity purification (TRAP) followed by RNA-seq (TRAP-seq) [17]. This study revealed the enrichment of *Ghrh* mRNA in hypothalamic LepRb neurons [17]. Leptin modulates food intake, glucose homeostasis, and linear growth [2], and *Ghrh* neurons also likely participate in the control of these parameters [18], [19], [20]. We therefore postulated that direct leptin action on LepRb^Ghrh^ neurons might mediate these effects.

To test this notion, we generated a knock-in mouse line to cotranslationally express Cre recombinase with *Ghrh* mRNA (*Ghrh*^*Cre*^ mice) (Figure 1A). Breeding *Ghrh*^*Cre*^ onto the Cre-dependent *Rosa26*^*eGFP-L10a*^ reporter background (Ghrh^eGFP−L10a^ mice) revealed the presence of Cre-expressing neurons in the expected areas of the hypothalamus, including the ARC (Figure 1B–D) [21], as well as in a few regions in the midbrain and hindbrain (Fig. S1). While the hypothalamic distribution of eGFP-L10a neurons mirrored the known adult expression pattern of *Ghrh* [21], the adult midbrain and hindbrain express little detectable *Ghrh*, suggesting either early developmental *Ghrh* expression in these regions or low-level expression in adults.

**Figure 1:**
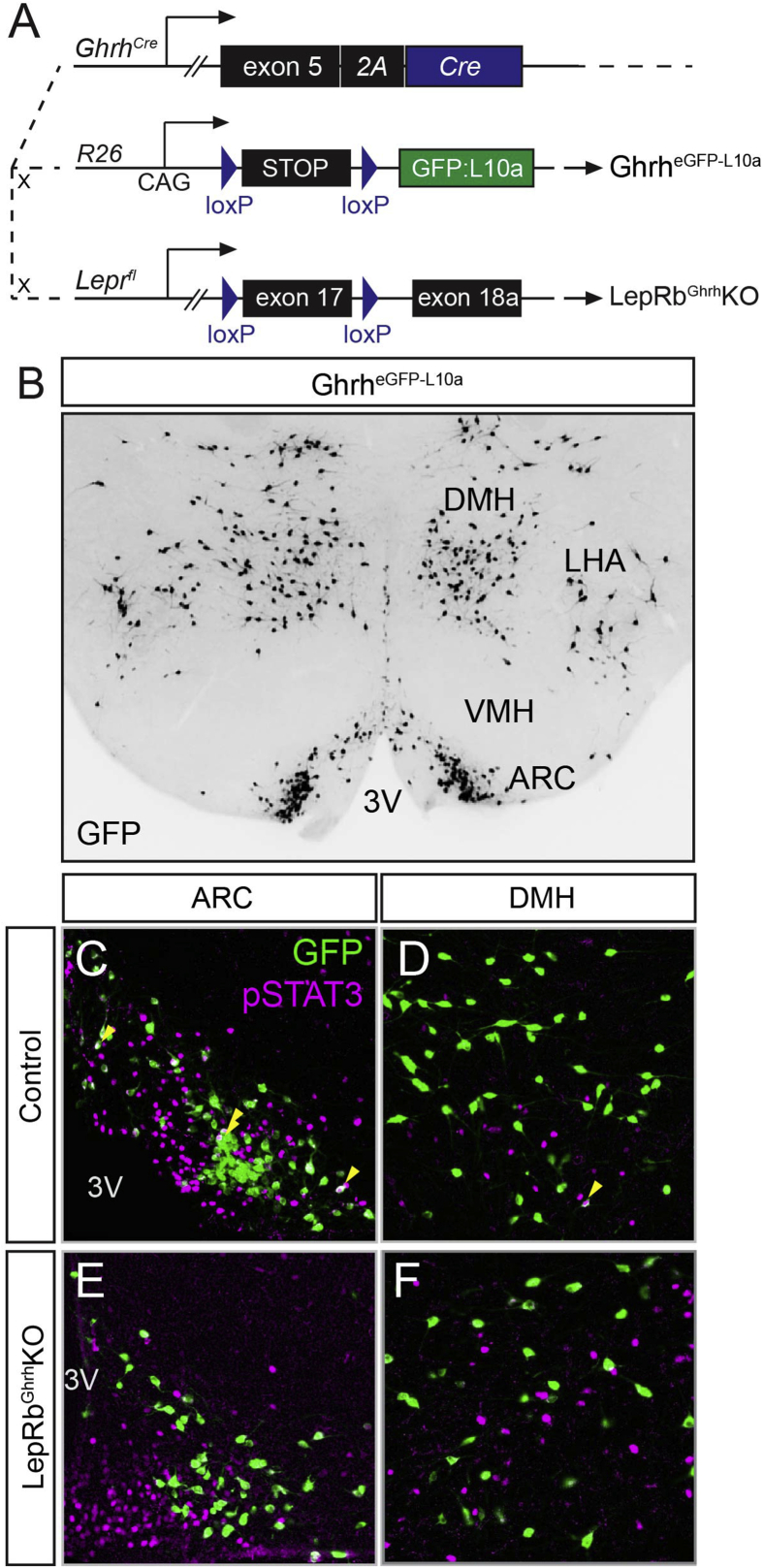
***Ghrh***^***Cre***^**mouse line**. (A) Diagram of mouse models used to manipulate *Ghrh* neurons. (B) Representative image of GFP IHC in the hypothalamus of Ghrh^eGFP−L10a^ mice. (C–F) Detection of leptin-stimulated pSTAT3 (magenta) and GFP (green) in Ghrh^eGFP−L10a^ (Control) and Lepr^Ghrh^KO mice on the *Rosa26*^*eGFP-L10a*^ background. Yellow arrowheads indicate examples of colocalized neurons. 3 V, third cerebral ventricle; all other abbreviations as defined in the text.

Single-cell sequencing of ARC neurons identified *Ghrh* neurons as distinct from AgRP and POMC neurons [22]. Similarly, *Ghrh*^*Cre*^ neurons are more lateral to the third ventricle than AgRP neurons [23] and do not colocalize with the laterally localized POMC neurons (Figs. S1E and F). Thus, our manipulation of *Ghrh*^*Cre*^ neurons will not directly impact AgRP or POMC neurons.

To identify LepRb^Ghrh^ neurons, we examined the detection of leptin-stimulated phosphorylated STAT3 (pSTAT3)-immunoreactivity (IR) and eGFP in Ghrh^eGFP−L10a^ mice by immunohistochemistry (IHC) (Figure 1C–D). The IHC detection of pSTAT3-IR reveals cell-autonomous leptin action on LepRb-expressing neurons [24]. As previously reported, no pSTAT3-immunoreactivity is detected in the brain in the absence of leptin action in *ob/ob* or *db/db* mice, and exogenous leptin promotes pSTAT3 in many hypothalamic cells in a distribution consistent with LepRb neurons (Fig. S2); previous results have demonstrated the colocalization of leptin-stimulated pSTAT3 with LepRb neurons [17]. Our analysis in Ghrh^eGFP−L10a^ mice revealed extensive colocalization of pSTAT3-IR and eGFP in the ARC (∼45% of ARC eGFP neurons were pSTAT3 positive). LepRb^Ghrh^ neurons represent a minority of total pSTAT3 neurons in the ARC, however. We also observed sparse colocalization in the DMH, but none in other areas, including the midbrain and hindbrain (data not shown).

To determine the importance of LepRb^Ghrh^ neurons to leptin action, we crossed *Ghrh*^*Cre*^ onto the *Lepr*^*flox*^ background to generate *Ghrh*^*Cre/+*^;*Lepr*^*fl/fl*^ (LepRb^Ghrh^KO) and littermate control (*Lepr*^*fl/fl*^) mice for study. Leptin treatment failed to promote the accumulation of pSTAT3 in eGFP-containing neurons in LepRb^Ghrh^KO mice on the *Rosa26*^*eGFP-L10a*^ background (Figure 1E,F), consistent with the ablation of LepRb from *Ghrh*^*Cre*^ neurons in these animals.

We examined the body weight and composition of LepRb^Ghrh^KO mice, as well as of mice with LepRb deleted in ARC POMC or AgRP neurons (LepRb^Pomc^KO and LepRb^Agrp^KO mice, respectively) (Figure 2). This analysis recapitulated the small (2–3 g) increase in body weight previously observed [7], [8] in both male and female LepRb^Pomc^KO and LepRb^Agrp^KO mice (Figure 2C,E). The increase in body weight in these animals reflected a tendency toward increased adiposity, except in the case of male LepRb^Pomc^KO mice, in which the increase in body weight reflected increased lean mass (Figure 2D,F). In contrast to the LepRb^Pomc^KO and LepRb^Agrp^KO mice, however, LepRb^Ghrh^KO mice exhibited no difference in body weight or body composition compared to controls (Figure 2A,B). Serum leptin and insulin concentrations were indistinguishable from controls for all three lines (Figure 3). Furthermore, we observed no alteration in food intake, blood glucose, or body length in male or female LepRb^Ghrh^KO mice compared to their controls (Fig. S2). Thus, direct leptin action via LepRb^Ghrh^ neurons is not required for the control of energy balance, glucose homeostasis, or linear growth in male or female mice, and leptin must control these parameters via other LepRb neurons. Thus, the early developmental ablation of LepRb from all previously-examined populations of ARC LepRb cells fails to substantially alter energy balance. Hence, the LepRb neurons not subject to developmental compensation that are most crucial for the control of energy balance either lie elsewhere in the hypothalamus or are redundant and distributed across multiple hypothalamic cell populations.

**Figure 2:**
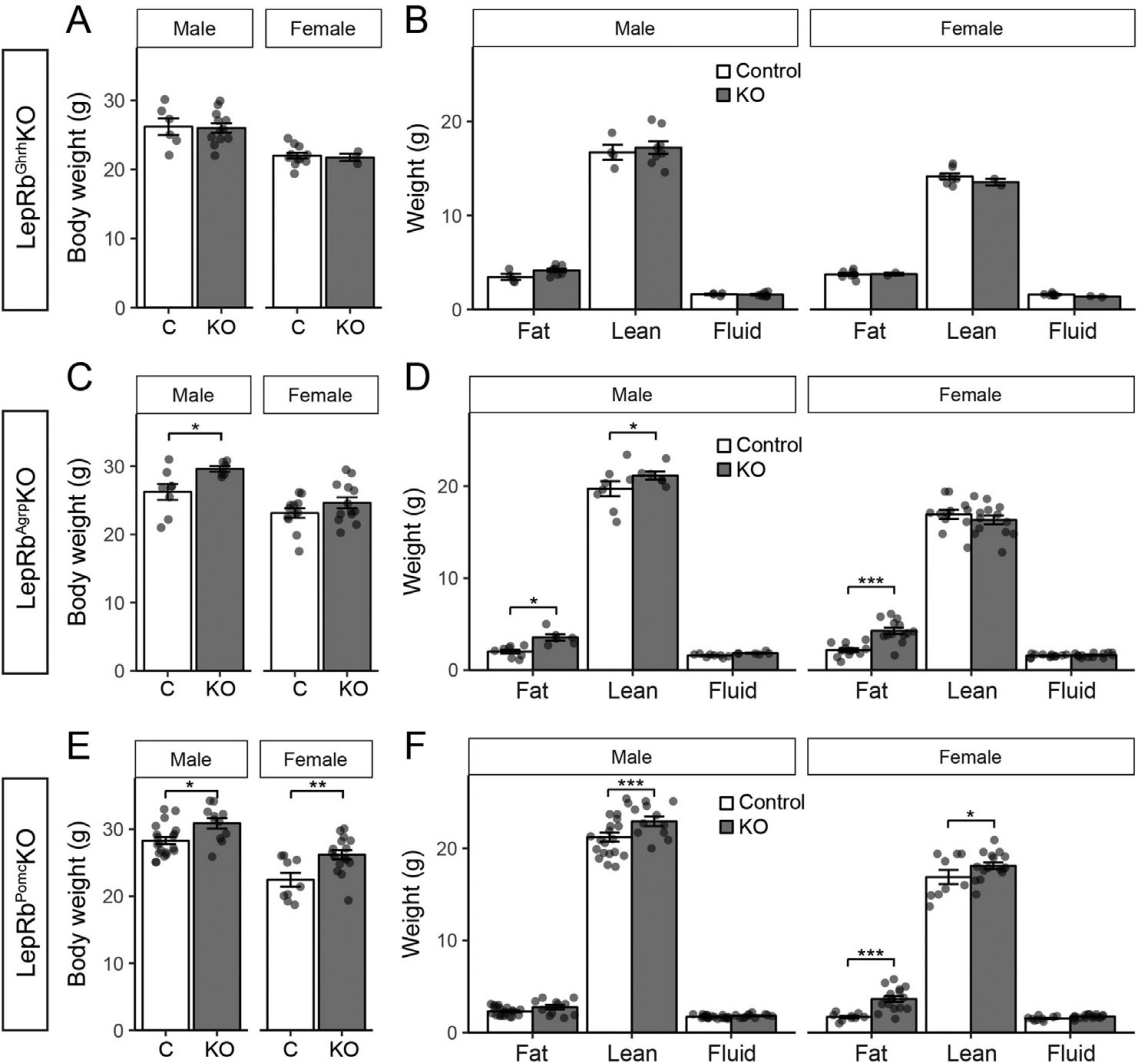
**Normal body weight and composition in LepRb**^**Ghrh**^**KO mice**. (A) Body weight in 12–13-week old male (n = 6 Control, 12 LepRb^Ghrh^KO) and female (n = 11 Control, 3 LepRb^Ghrh^KO) mice. (B) 13-week old body composition in male (n = 4 Control, 7 LepRb^Ghrh^KO) and female (n = 8 Control, 2 LepRb^Ghrh^KO) mice. (C) Body weight in 8-week old male (n = 8 Control, 6 LepRb^Agrp^KO) and female (n = 12 Control, 13 LepRb^Agrp^KO) mice. Significance determined by one-way ANOVA with Tukey's post-test. (D) 8-week old body composition in male (n = 8 Control, 6 LepRb^Agrp^KO) and female (n = 12 Control, 13 LepRb^Agrp^KO) mice. Significance determined by linear mixed model with Tukey's post-test. (E) Body weight in 8-week old male (n = 20 Control, 11 LepRb^Pomc^KO) and female (n = 9 Control, 16 LepRb^Pomc^KO) mice. Significance determined by one-way ANOVA with Tukey's post-test. (F) 8-week old body composition in male (n = 20 Control, 11 LepRb^Pomc^KO) and female (n = 9 Control, 16 LepRb^Pomc^KO) mice. Significance determined by linear mixed model with Tukey's post-test.

**Figure 3:**
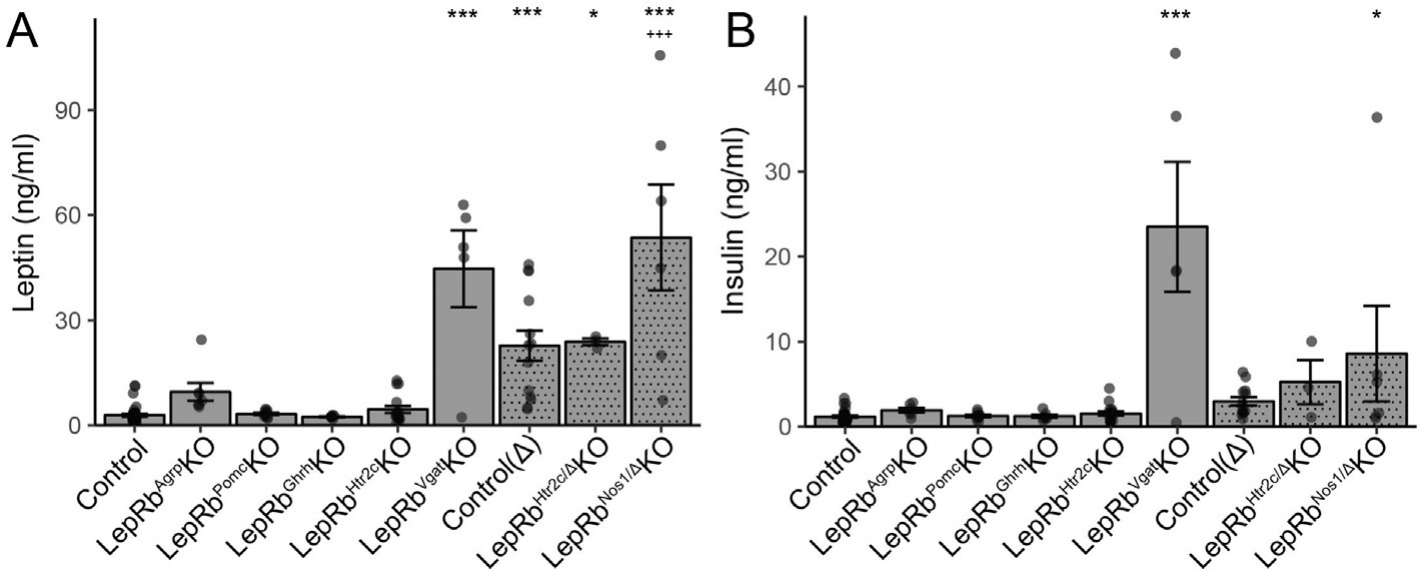
**Leptin and insulin levels in various LepRb mutant mouse line**. (A–B) Leptin (A) and insulin (B) levels in the indicated mutant mouse lines. Significance determined by one-way ANOVA with Sidak's post-test: * indicate significance against Control and + indicate significance against Control (Δ).

### 2.2 The role for widely-distributed LepRb^Htr2c^ neurons in leptin action

We previously generated knock-in mice to express Cre recombinase from the *Htr2c* locus (*Htr2c*^*Cre*^ mice) [25]; breeding these to the *Rosa26*^*eGFP-L10a*^ reporter strain (Htr2c^eGFP−L10a^ mice) (Figure 4A) revealed the distribution of *Htr2c* neurons across many hypothalamic regions (Figure 4B–D). Furthermore, the detection of leptin-stimulated pSTAT3 in Htr2c^eGFP−L10a^ mice revealed substantial colocalization of pSTAT3 with eGFP in the PMv, VMH, LHA, ARC, and DMH; we observed no colocalization outside of the hypothalamus, however (data not shown). Thus, LepRb^Htr2c^ neurons represent a broadly-distributed population of hypothalamic LepRb neurons.

**Figure 4:**
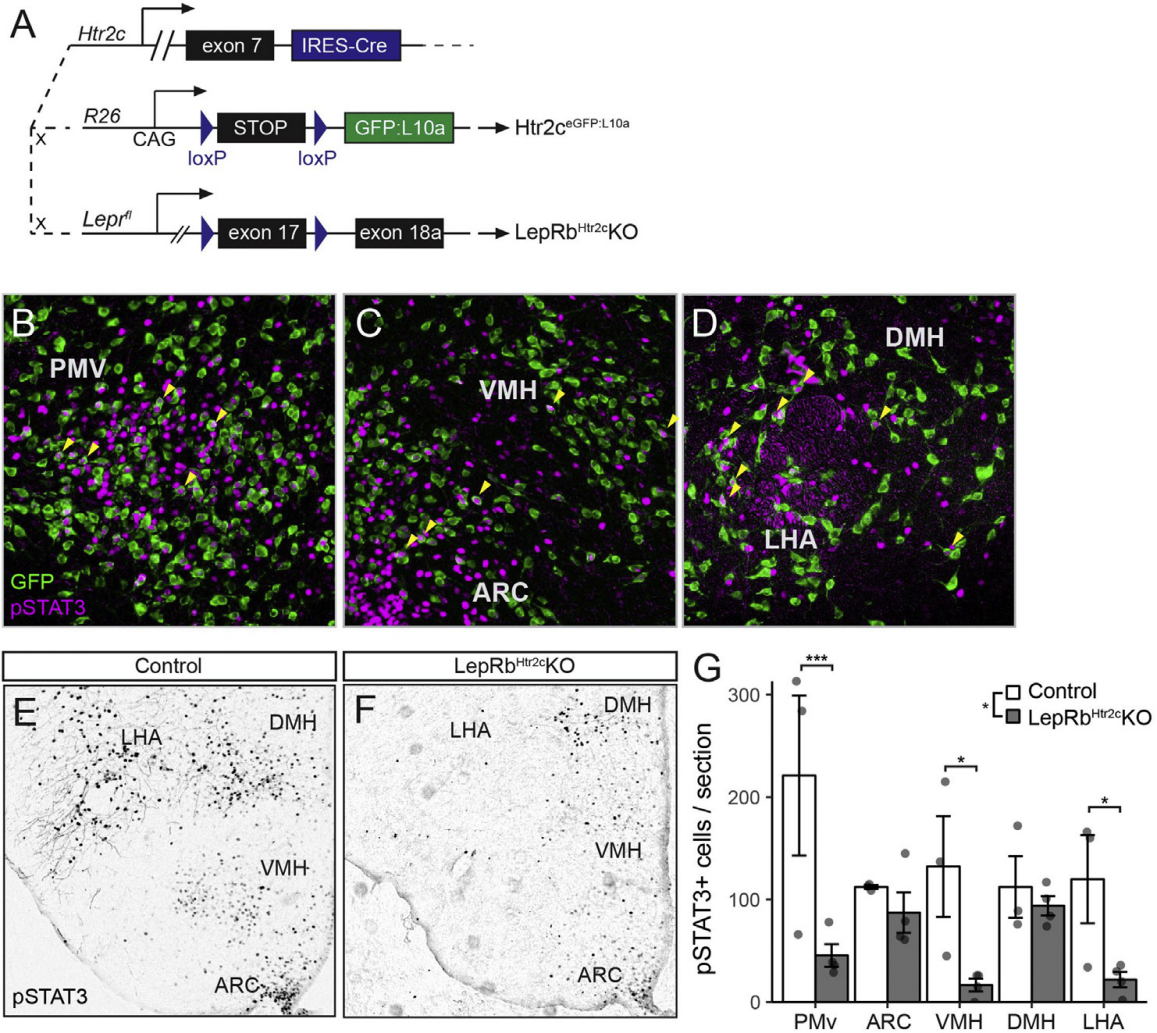
**Hypothalamic distribution of LepRb**^**Htr2c**^**neurons**. (A) Diagram of mouse models used to manipulate *Htr2c* neurons. (B–D) Representative images showing colocalization of leptin-stimulated pSTAT3 (magenta) and Htr2c^eGFP−L10a^ (GFP, green) in multiple hypothalamic nuclei. (E–F) pSTAT3 staining in Control (n = 3) and LepRb^Htr2c^KO mice (n = 4); quantification of pSTAT3 by nucleus is shown in (G).

To test whether the total number of hypothalamic LepRb neurons subjected to early developmental LepRb ablation dictates the disruption of energy balance, we bred *Htr2c*^*Cre*^ onto the *Lepr*^*fl*^ background to generate *Htr2c*^*Cre*^;*Lepr*^*fl/fl*^ (LepRb^Htr2c^KO) and littermate control (*Lepr*^*fl/fl*^) mice. Comparing the detection of leptin-stimulated pSTAT3 in the hypothalamus of LepRb^Htr2c^KO and control mice revealed the almost complete ablation of pSTAT3/LepRb from the PMv, VMH, and LHA (all three areas p < 0.05), with more modest reductions of pSTAT3 in the ARC and DMH (Figure 4E–G). While *Htr2c* is expressed in POMC neurons [25], the POMC subpopulation that expresses *Htr2c* is distinct from that which expresses *Lepr* [26], [27]. Thus, the few LepRb^Htr2c^ neurons in the ARC do not include POMC cells. Overall, LepRb^Htr2c^KO mice display ∼50% loss of hypothalamic pSTAT3.

**Figure 5:**
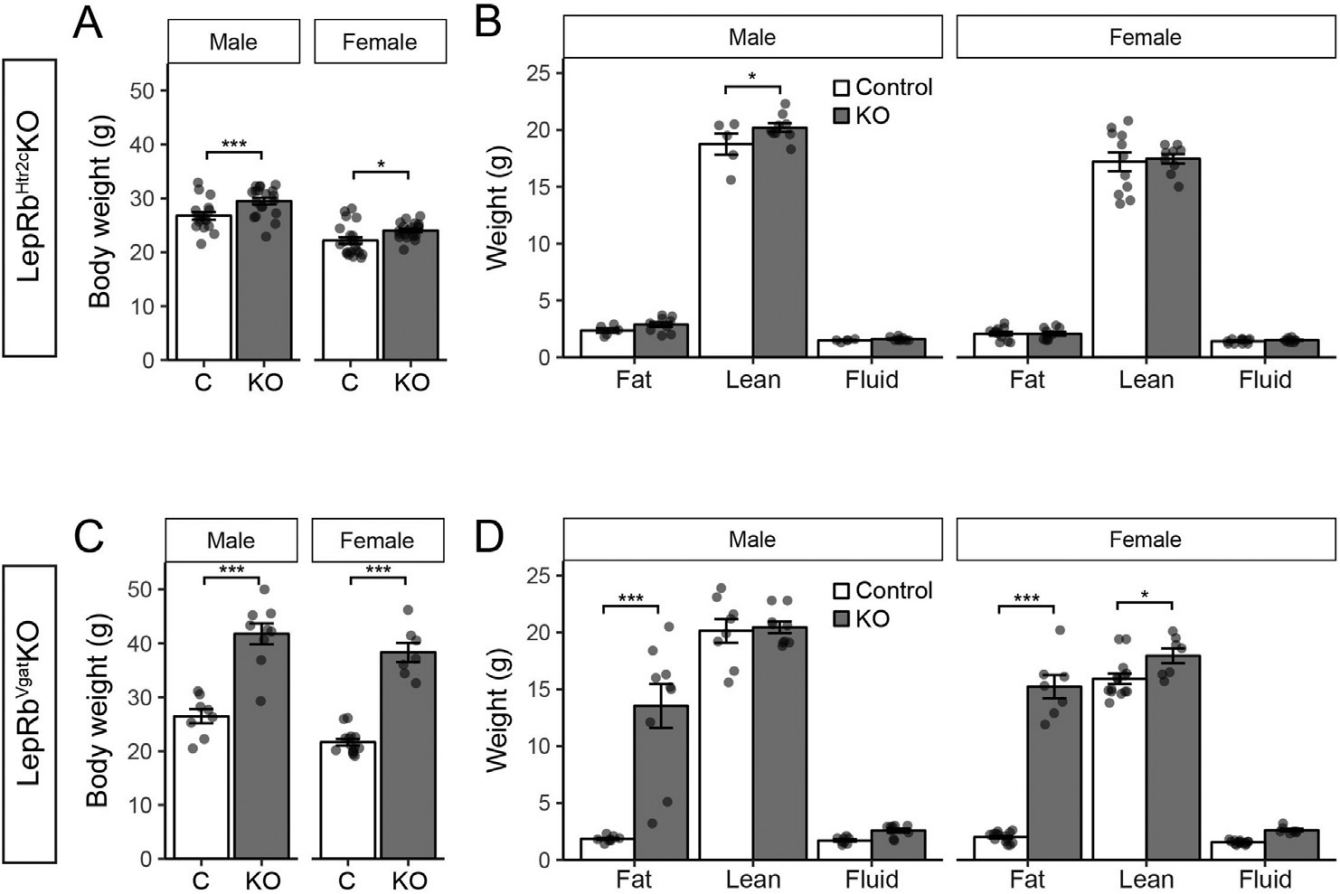
**Increased lean mass in LepRb**^**Htr2c**^**KO mice**. (A) Body weight in 8-week old male (n = 17 Control, 19 LepRb^Htr2c^KO) and female (n = 22 Control, 20 LepRb^Htr2c^KO) mice. Significance determined by one-way ANOVA with Tukey's post-test. (B) 8-week old body composition in male (n = 5 Control, 9 LepRb^Htr2c^KO) and female (n = 11 Control, 9 LepRb^Htr2c^KO) mice. Significance determined by linear mixed model with Tukey's post-test. (C) Body weight in 8-week old male (n = 8 Control, 9 LepRb^Vgat^KO) and female (n = 14 Control, 7 LepRb^Vgat^KO) mice. Significance determined by one-way ANOVA with Tukey's post-test. (D) 8-week old body composition in male (n = 8 Control, 9 LepRb^Vgat^KO) and female (n = 14 Control, 7 LepRb^Vgat^KO) mice. Significance determined by linear mixed model with Tukey's post-test.

We compared the body weight phenotype of LepRb^Htr2c^KO mice to that of mice ablated for LepRb in vGat neurons (LepRb^vGat^KO mice), in which LepRb is ablated from the majority of LepRb neurons in the ARC, DMH and LHA [12]. While LepRb^Htr2c^KO mice of both sexes were slightly heavier than littermate controls (Figure 5A), their increased body weight was small relative to the dramatic reduction in their number of hypothalamic LepRb cells and arose mainly from increased lean mass, not fat mass (Figure 5B). In contrast and as previously reported [12], LepRb^vGat^KO mice of both sexes displayed dramatically increased body weight and adiposity (Figure 5C,D; Fig. S3A). Furthermore, LepRb^Htr2c^KO mice exhibited neither hyperglycemia (Fig. S3B) nor alteration in serum insulin or leptin concentrations (Figure 3). Thus, ablating ∼50% of hypothalamic LepRb neurons in LepRb^vGat^KO mice dramatically produced obesity, while ablating similar number of hypothalamic LepRb neurons in LepRb^Htr2c^KO mice failed to increase adiposity, demonstrating that the specific LepRb cell type affected matters more than the total number of hypothalamic neurons from which LepRb is ablated.

**Figure 6:**
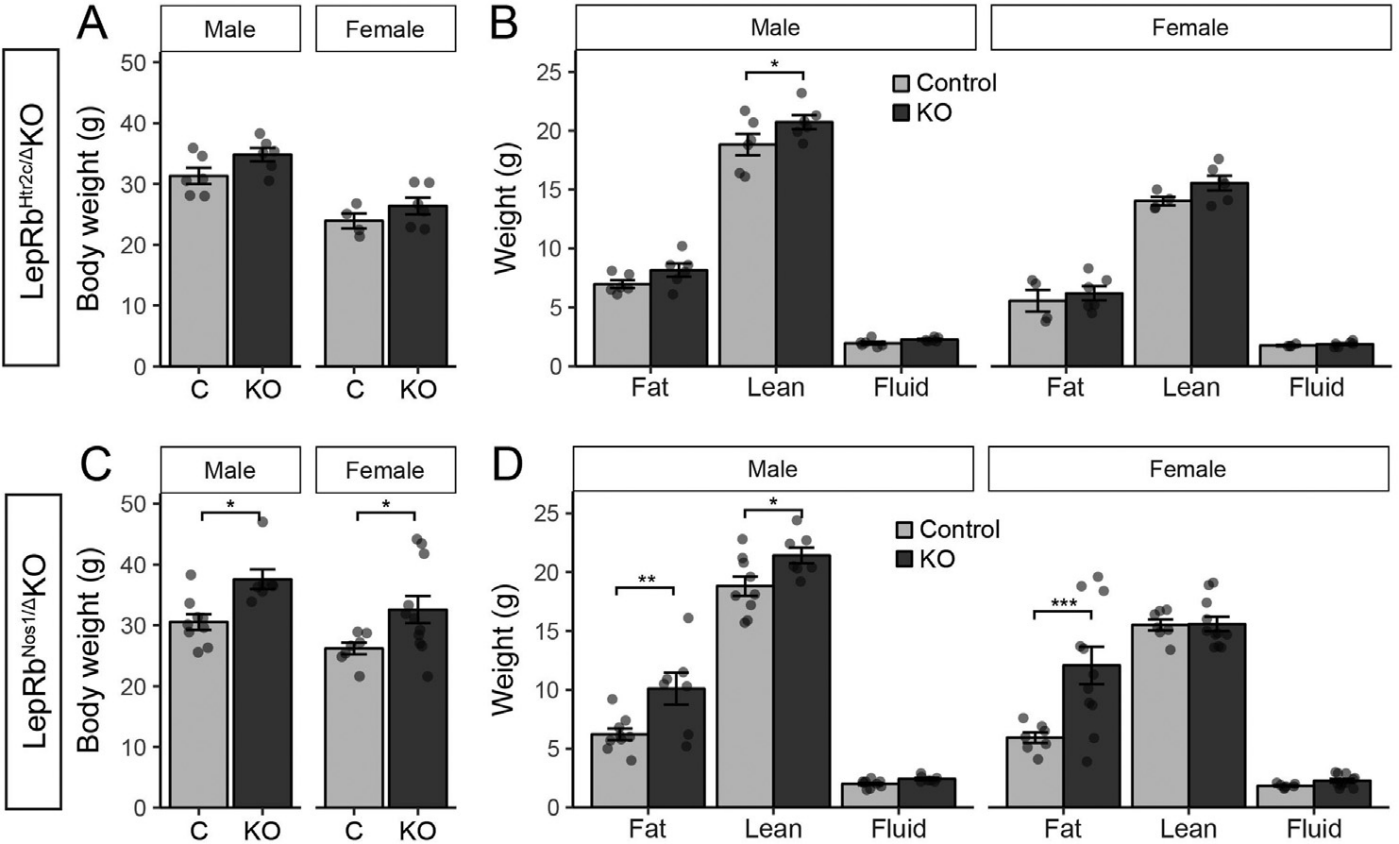
**Increased lean mass in LepRb**^**Htr2c/Δ**^**KO mice**. (A) Body weight in 8-week old male (n = 6 Control, 6 LepRb^Htr2c/Δ^ KO) and female (n = 4 Control, 6 LepRb^Htr2c/Δ^ KO) mice. Significance determined by one-way ANOVA with Tukey's post-test. (B) 8-week old body composition in male (n = 6 Control, 6 LepRb^Htr2c/Δ^ KO) and female (n = 4 Control, 6 LepRb^Htr2c/Δ^ KO) mice. Significance determined by linear mixed model with Tukey's post-test. (C) Body weight in 8-week old male (n = 9 Control, 7 LepRb^Nos1/Δ^ KO) and female (n = 7 Control, 11 LepRb^Nos1/Δ^ KO) mice. Significance determined by one-way ANOVA with Tukey's post-test. (D) 8-week old body composition in male (n = 9 Control, 7 LepRb^Nos1/Δ^ KO) and female (n = 7 Control, 11 LepRb^Nos1/Δ^ KO) mice. Significance determined by linear mixed model with Tukey's post-test.

We previously discovered a role for LepRb^Nos1^ neurons in the control of energy balance by leptin [13]. Due to the high frequency of *Nos1*^*Cre*^-mediated excision of *Lepr*^*fl*^ (producing *Lepr*^*Δ*^) in the female germline, we studied *Nos1*^*Cre/+*^*;Lepr*^*Δ/fl*^ (LepRb^Nos1/Δ^KO) compared to littermate *Lepr*^*Δ/fl*^ or *Lepr*^*Δ/+*^ (LepRb^Δ/?^) control animals. It is theoretically possible that the ablation of 50% of LepRb expression in all neurons sensitized the phenotype of LepRb^Nos1/Δ^KO; indeed, *Lepr*^*Δ/+*^ mice exhibit a mild increase in body weight and adiposity compared to *Lepr*^*+/+*^ or *Lepr*^*fl/fl*^ animals (see, for example leptin levels in Figure 3). We thus generated *Htr2c*^*Cre/+*^*;Lepr*^*Δ/fl*^ (LepRb^Htr2c/Δ^KO) and littermate *Lepr*^*Δ/+*^ or *Lepr*^*Δ/fl*^ controls for study; we also generated LepRb^Nos1/Δ^KO and littermate controls for comparison (Figure 6).

While LepRb^Htr2c/Δ^KO mice of both sexes tended to weigh more than their controls, the difference was not statistically significant (Figure 6). Furthermore, as for LepRb^Htr2c^KO mice without a *Lepr*^*Δ*^ allele, male mice exhibited an increase in lean mass, rather than adiposity, and leptin and insulin concentrations did not differ from controls (Figure 3). In contrast, LepRb^Nos1/Δ^KO mice of both sexes exhibited increased body weight and adiposity (Figure 6C,D), along with increased circulating leptin and insulin (Figure 3), as previously reported. Thus, the presence of a *Lepr*^*Δ*^ allele failed to unmask an energy balance phenotype due to the ablation of LepRb in Htr2c neurons, confirming that the LepRb^Htr2c^ cells play little role in the control of energy balance by leptin.

## 3 Discussion

Despite the passing of more than two decades since the discovery of leptin [4], the cellular mediators (i.e., LepRb neurons) that mediate the largest component of the dramatic obesity phenotype of *db/db* mice remain undefined. To date, over a dozen anatomically-circumscribed populations of cells have been identified as leptin-responsive, but all of those studied by early developmental ablation of LepRb play only small or negligible roles in the control of energy balance by leptin [7], [8], [9], [10], [11], [17]. Here, we demonstrate that ARC *Ghrh*^*cre*^ neurons are leptin responsive, but are also dispensable for leptin action.

It is important to note that our analysis (like most others) of the phenotypes derived from LepRb ablation in specific cell types rests on the analysis of mice in which the LepRb ablation occurs at an early developmental stage [16], rendering developmental compensation/remodeling of neural circuits to mask potential phenotypes. Indeed, while manipulation of AgRP neurons in adults (including LepRb deletion) promotes dramatic changes in energy balance [14], [15], [16], early developmental alterations in AgRP neurons produce minimal phenotypes as a consequence of developmental compensation [7], [15]. Thus, our analysis focuses on the circuits whose dysfunction cannot be compensated in this manner.

The failure to alter energy balance upon early developmental LepRb ablation in most cell types might be explained by the distribution of leptin action across multiple populations, such that each population alone is dispensable. Indeed, the findings that LepRb deletion from broadly distributed hypothalamic populations (*vGat* or *Nos1*) dramatically increases food intake, body weight and fat mass [12], [13] supports this distributed model. Here, we explicitly tested this model by removing leptin action from the large broadly-distributed population of hypothalamic LepRb^Htr2c^ neurons. Ablation of LepRb from *Htr2c* cells failed to substantially alter energy balance or parameters of glucose homeostasis – even when bred onto a potentially sensitizing *Lepr*^*Δ*^ background. Indeed, if anything, the slight phenotype of *Lepr*^*Δ*^ decreases the ability to detect alterations in weight gain by neuron-specific LepRb ablation.

Thus, LepRb^Htr2c^ neurons play a minimal role in the control of energy balance by leptin, and deletion of LepRb from approximately half of hypothalamic LepRb neurons does not necessitate obesity if the important cells are not impacted. Additionally, some set of non-*Htr2c*, non-AgRP, non-POMC, non-*Ghrh* cells must be especially important for the *db/db* phenotype.

### 3.1 Potentially crucial role for DMH LepRb neurons in the control of energy balance

*Htr2c*^*Cre*^ deletes LepRb mainly in the PMv, VMH, and LHA (but minimally impacts the ARC and DMH) and promotes no adiposity phenotype, while *Nos1*^*Cre*^ (which deletes LepRb in most of the PMv plus 5–15% of cells in the ARC, DMH, VMH and LHA) and *vGat*^*Cre*^ (which deletes LepRb in the ARC, DMH, and LHA) both produce substantial obesity [12], [13]. These data suggest that direct leptin action via LepRb neurons in the PMv, VMH, and LHA play minimal roles in the *db/db* phenotype, consistent with the minimal phenotypes produced by modulation of LepRb in the PMv [28], in the VMN [11], in vGlut2 cells [12] (PMv plus VMH and many brainstem neurons), and in *Nts*-expressing LHA LepRb neurons [10].

Thus, leptin action via LepRb neurons in the ARC and/or DMH must play a predominant role in the phenotype of *db/db* mice. Since *Nos1*^*Cre*^-mediated LepRb ablation results in substantial obesity [13], but relatively few LepRb^Nos1^ neurons lie in the ARC and DMH, the crucial populations of LepRb neurons must be relatively small. Furthermore, because ablation of LepRb in ARC AgRP, POMC, or *Ghrh*^*Cre*^ cells minimally alters energy balance, DMH LepRb neurons likely play an especially important role in the *db/db* phenotype [29].

Consistent with the small number of LepRb^Nos1^ neurons that lie in the DMH (but presumably contribute to the phenotype of LepRb^Nos1/Δ^KO mice), not all DMH LepRb neurons appear to play a substantial role in the control of energy balance. For instance, ablation of LepRb from DMH prolactin-releasing hormone (*Prlh*) neurons produces a small phenotype that results from altered energy expenditure, rather than dysregulated food intake [9]. Similarly, ablation of LepRb in prodynorphin neurons (which ablates 43% of DMH LepRb) produces no detectable body weight phenotype [17]. Thus, the small number of DMH LepRb^Nos1^ cells (presumably those that overlap with DMH LepRb^vGat^ neurons) likely represent crucial controllers of body energy homeostasis. More clearly defining the molecular phenotype of DMH LepRb cells that contribute to the phenotype of early developmental LepRb deficiency and that are crucial for the control of energy balance represents an important goal of future research.

## 4 Materials and methods

### 4.1 Animals

All procedures performed on animals were approved by the University of Michigan Institutional Animal Care and Use Committee and in accordance with AAALAC and NIH guidelines. All mice were bred in our colony in the Unit for Laboratory Animal Management at the University of Michigan. All mice were provided with water *ad libitum* and housed in temperature-controlled rooms on a 12-hour/12-hour light–dark cycle. All mice were provided *ad libitum* access to standard chow diet (Purina Lab Diet 5001).

*Rosa26*^*eGFP-L10a*^ [17], *Lepr*^*flox*^ [30], *Htr2c*^*Cre*^ [25], *Agrp*^*Cre*^ (Jax 012899) [23], *Pomc-Cre* (Jax 005965) [8], *Nos1*^*Cre*^ (Jax 017526) [13], and *vGat*^*Cre*^ (Jax 016962) [12] mice have been previously described. All mice were weaned at 21 of age and group-housed with littermates of the same sex unless otherwise stated.

#### 4.1.1 Ghrh^Cre^ generation

To generate *Ghrh*^*Cre*^ mice, a selection cassette containing the porcine teschoviral 2 A cleavage sequence linked to Cre recombinase and a FRT-flanked neomycin resistance gene was targeted to replace the stop codon of the *Ghrh* gene in a bacterial artificial chromosome (Children's Hospital Oakland Research Institute). A targeting plasmid containing the Cre coding sequences plus the selection cassette and ∼4 kb genomic sequence upstream and downstream of the *Ghrh* stop codon was isolated and used for embryonic stem cell targeting by the University of Michigan Transgenic Core. Correctly targeted clones were identified by loss of native allele quantitative PCR from ES clone DNA. Chimeric animals generated from blastocyst implantation were then bred for germline transmission of the *Ghrh*^*Cre*^ allele. Flp-deleter mice were then used to remove the neomycin selection cassette. Genotyping was by allele-specific PCR.

### 4.2 Longitudinal study

All Cre mouse lines were crossed several times to *Lepr*^*fl/fl*^ mice to obtain breeders to generate study mice. For most lines, *Cre/*+*;Lepr*^*fl/fl*^ mice were bred to *Lepr*^*fl/fl*^ mice such that roughly half of all animals would be mutants and the other half would be littermate controls. *Htr2c* resides on the Y chromosome, so only female *Htr2c*^*Cre*^ breeders were used, and all study males were *Htr2c*^*Cre/y*^;*Lepr*^*fl/fl*^ hemizygotes, while all females were *Htr2c*^*Cre/+*^;*Lepr*^*fl/fl*^. For *vGat*^*Cre*^, however, *vGat*^*Cre/+*^*;Lepr*^*fl/+*^ mice were bred to *Lepr*^*fl/fl*^ to generate *Lepr*^*fl/fl*^ and *vGat*^*Cre/+*^*;Lepr*^*fl/fl*^ mice. For *Nos1*^*Cre*^, *Nos1*^*Cre*^*;Lepr*^*Δ/+*^ males were crossed to *Lepr*^*fl/fl*^ females to generate *Lepr*^*Δ/+*^ and *Nos1*^*Cre*^*;Lepr*^*Δ/fl*^ mice for study; we generated *Htr2c*^*Cre*^*;Lepr*^*Δ/fl*^ similarly.

LepRb^Ghrh^KO mice were single-housed with enrichment at 4 weeks old to measure continuous food intake. Body weight and food weight were measured weekly; blood glucose (from tail vein bleeds; OneTouch Ultra 2) and snout-anus length were measured biweekly. For snout-anus length, mice were briefly anesthetized with isoflurane and gently stretched on their back while calipers (Scienceware) measured the snout-anus distance. At 12-weeks of age, animals were subjected to NMR-based (Minispec LF90ll, Bruker Optics) body composition analysis. Prior to euthanasia, serum was obtained from some animals for the determination of leptin and insulin by commercial ELISA (Crystal Chem).

For all other mouse lines (*Agrp*^*Cre*^, *Pomc-Cre*, *vGat*^*Cre*^, *Nos1*^*Cre*^, *Htr2c*^*Cre*^), animals were group-housed until age 8 weeks, at which point they were weighed and subjected to body composition analysis with subsequent serum collection. Additionally, we subjected a separate cohort of LepRb^Htr2c^KO mice and their littermate controls to weekly body weight and biweekly blood glucose measurements.

### 4.3 Immunohistochemistry (IHC)

For the detection of pSTAT3, mice had food removed at the end of the light cycle; the next morning, mice were treated with murine leptin IP (5 mg/kg) (the generous gift of MedImmune, Inc.) for 1–2 h. These and all other mice for immunohistochemistry analysis were anesthetized with either isoflurane or tribromoethanol (Avertin) and transcardially perfused with phosphate buffered saline (PBS) followed by 10% buffered formalin. Brains were removed, placed in 10% buffered formalin overnight, and cryoprotected in 30% sucrose for several days. Using a freezing microtome (Leica), brains were cut into 30 μm sections. Sections were treated sequentially with 1% hydrogen peroxide/0.5% sodium hydroxide, 0.03% sodium dodecyl sulfate, and blocking solution (PBS with 0.3% Triton, 3% Normal Donkey Serum). Immunostaining was performed using primary antibodies for pSTAT3 (Cell Signaling #9145, 1:500), GFP (Aves Labs #GFP1020, 1:1000), and POMC (Phoenix Pharmaceuticals #H-029-30, 1:5000). GFP antibodies were reacted with species-specific Alexa Fluor-488 secondary antibody (Invitrogen, 1:250), POMC antibodies with Alexa Fluor-568 secondary antibody (Invitrogen, 1:250), and pSTAT3 antibodies were processed with the avidin-biotin/diaminobenzidine (DAB) method (ABC kit, Vector Labs, 1:200; DAB reagents, Sigma). Images were collected on an Olympus BX51 microscope with Olympus DP80 camera. DAB images were pseudocolored using FIJI software (http://fiji.sc/). FIJI was also used for image quantification using the Cell Counter plugin.

### 4.4 Statistics

Data are reported as mean ± SEM; additionally, the values of all replicates are shown when feasible. Data analysis was performed in R 3.4.3. Body weight comparisons, plus insulin and leptin ELISA data, were analyzed with a one-way ANOVA with Sidak's multiple comparisons correction. Body composition analysis was conducted using a linear mixed model with fixed effects of genotype, sex, and component (fat, lean, fluid) and random effects of mouse. Longitudinal studies were conducted using a linear mixed model with fixed effects of age, sex, and, genotype and random effects of mouse. All p values from estimated marginal means calculated from linear mixed models were corrected using Tukey's multiple comparisons correction. Linear mixed models were conducted with lme4 1.1–15, lmerTest 2.0–36, and emmeans 1.1.2. Star code on graphs: *p < 0.05, **p < 0.01, ***p < 0.001.

## Author contributions

ACR, MBA, JCJ, CMP, CLF, LKH, DPO, and MGM designed experiments; ACR, MBA, JCJ, CMP, CLF, NB, and DPO researched data. ACR and MGM prepared figures and wrote the initial manuscript draft. All authors edited the manuscript.

## Figures and Tables

**FigS1:**
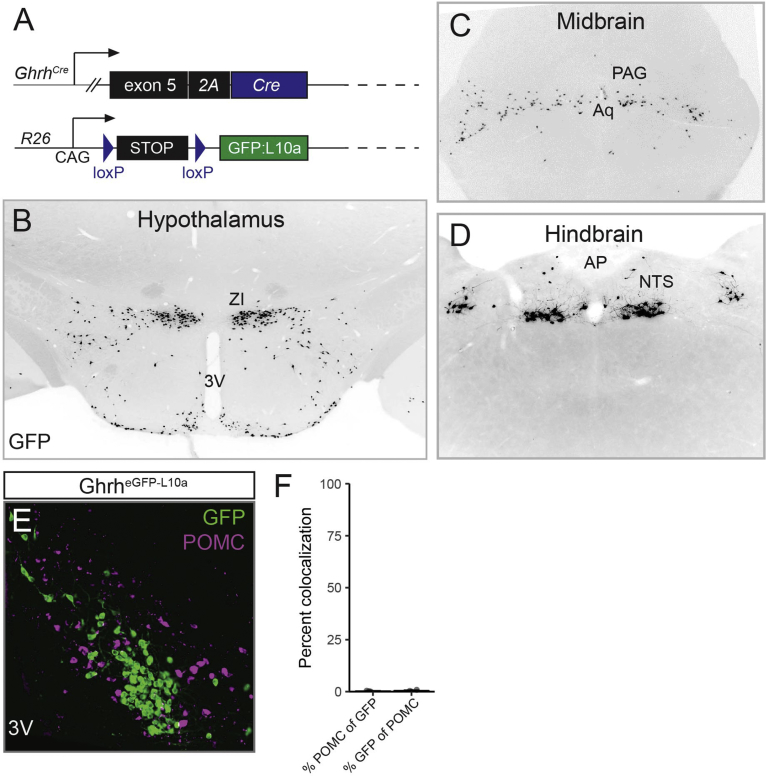
***Ghrh***^***Cre***^**reporter activity across multiple brain areas**. (A) Diagram of mouse models used to manipulate *Ghrh* neurons. (B–D) Representative images of GFP expression in the rostral hypothalamus, midbrain, and hindbrain of Ghrh^eGFP−L10a^ mice. (E) Co-staining for GFP (green) and POMC (magenta) in Ghrh^eGFP−L10a^ mice reveals no overlap between the two populations. (F) Quantification of GFP and POMC colocalization (n = 3 mice). Data are not significantly different from 0, p = 0.233 by one-sample t-test.

**FigS2:**
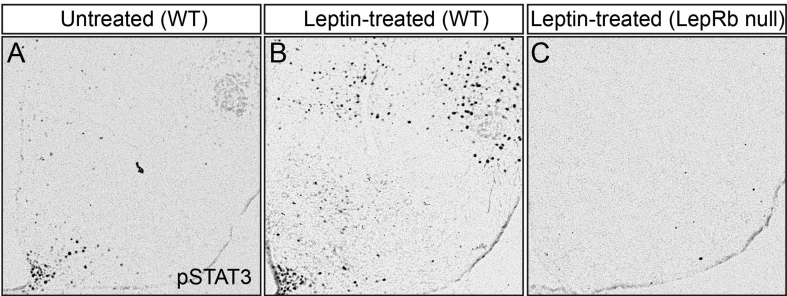
**Leptin-stimulated pSTAT3 staining in the hypothalamus**. Shown are representative images demonstrating pSTAT3 staining (black) in the hypothalamus of untreated wild-type (A) and leptin-treated (5 mg/kg, IP, 1 h) wild-type (B) or LepRb null (*Lepr*^*neo/neo*^) (C) mice.2

**FigS3:**
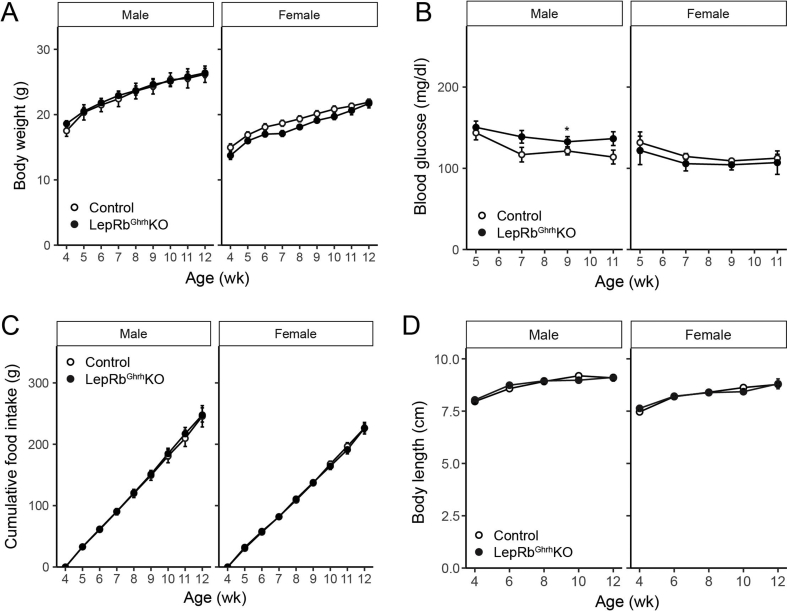
**LepRb**^**Ghrh**^**KO mice have normal body weight and blood glucose**. (A–D) Longitudinal study of Control (n = 6 male and 11 female) and LepRb^Ghrh^KO (n = 12 male and 3 female) mice. (A) Body weight, (B) blood glucose, (C) cumulative food intake, and (D) snout-anus length for Ghrh^eGFP−L10a^ from the time of weaning until 12 weeks of age. For all data, significance determined by linear mixed model with Tukey's post-test.3

**FigS4:**
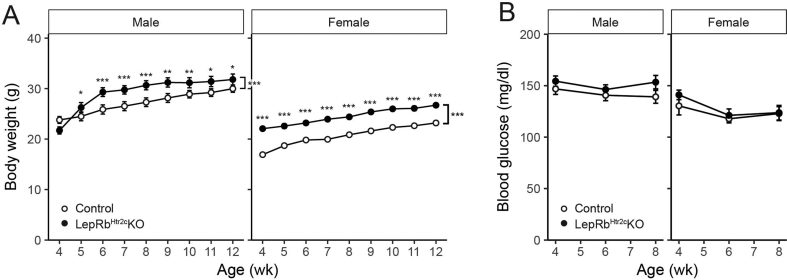
**LepRb**^**Htr2c**^**KO mice have mildly elevated body weight and normal blood glucose**. (A) Weekly body weight in Control (n = 9–11 male and 11 female) and LepRb^Htr2c^KO (n = 10–11 male and 11 female) mice. (B) Biweekly glucose measurement in Control (n = 10 male and 10 female) and LepRb^Htr2c^KO (n = 10 male and 10 female) mice. For both, significance determined by linear mixed model with Tukey's post-test.4
